# Comparison of clinical outcomes between laparoscopic orchiopexy and open orchiopexy in the treatment of palpable undescended testes

**DOI:** 10.12669/pjms.39.3.7371

**Published:** 2023

**Authors:** Zhuangzhuang Chen, Yansheng Chu, Yifeng Hu

**Affiliations:** 1 Zhuangzhuang Chen, Department of Pediatric Surgery, Zaozhuang Municipal Hospital, Zaozhuang 277100, Shandong Province, P.R. China; 2 Yansheng Chu, Department of Pediatric Surgery, Zaozhuang Yicheng District People’s Hospital, Zaozhuang 277300, Shandong Province, P.R. China; 3 Yifeng Hu, Department of Pediatric Surgery, Zaozhuang Municipal Hospital, Zaozhuang 277100, Shandong Province, P.R. China

**Keywords:** Laparoscopic orchiopexy, Open orchiopexy, Palpable undescended testes

## Abstract

**Objective:**

To compare the clinical effects of laparoscopic orchiopexy (LO) and open orchiopexy (OO) in the treatment of palpable undescended testes.

**Methods:**

Seventy-six children with palpable undescended testes treated in Zaozhuang Municipal Hospital from June 2019 to January 2021 were selected in this observational retrospective study. Patients were grouped according to their different surgical methods, 33 patients received OO (Open-group) and 43 patients received LO (Laparoscopic-group). The clinical outcomes of the two groups were compared, including surgical-related indicators, near and long-term surgical complications and postoperative testicular growth.

**Results:**

Operation time, intraoperative bleeding, first ambulation time and hospitalization time in the Laparoscopic-group were lower than those in the Open-group (p<0.05). The short-term complication rate in the Laparoscopic-group was lower than that in the Open-group (2.27% vs 15.15%; p<0.05), but the long-term complication rate in the Laparoscopic-group was not significantly different from the Open-group (4.65% vs 3.03%; p>0.05). Follow-up was up to 18 months post-operation, with the rate of testicular growth (97.67% vs 96.97%; p>0.05) and testicular volume (0.59 ± 0.14ml vs 0.58 ± 0.12ml p>0.05) not significantly different between the Laparoscopic-group or Open-group respectively.

**Conclusion:**

LO is as clinically effective as OO in the treatment of palpable undescended testes, however, shorter operation time, less intraoperative bleeding and rapid recovery time have been noted with LO.

## INTRODUCTION

Undescended testes are a common congenital malformation of the urinary system in children, and refers to unilateral or bilateral testicular loss caused by testicular growth that does not smoothly descend to the normal scrotum position through the abdominal cavity, but stays at the abnormal transitional site. The incidence is 2-4% in newborns and can increase up to 45% in premature infants.^1^,^2^ The occurrence of undescended testes can not only affect sperm development, maturation and reproductive function, but children with testicular failure have a higher risk of testicular malignancy.^3^ Moreover, the occurrence of undescended testes can have adverse effects on gonadal development, which is closely related to male infertility and testicular malignancy.

The undescended testicle typically remains in the abdomen while descended testes are found within the scrotum. The temperature within the scrotum is lower than that of the abdomen, and the elevated temperature can damage germ cells increasing the risk of malignant lesions.^4^ The testicles should drop by six months of birth, and studies have shown a higher rate of cancer in children treated after the age of 10. Therefore, it is necessary to treat undescended testes closely following diagnosis. Domestic and foreign guidelines point out that it is necessary to follow the principles of early diagnosis and treatment.^5^

Currently, treatment of undescended testes can include hormone and surgical treatment. Generally, hormone treatment has an efficacy of 20%. For those children whose testicles have not yet descended into the scrotum after one year of age, and hormone treatment is not effective, surgery is recommended to assist testicular descendance into the scrotum.^6^ Testicular fixation is the most used method for the treatment of undescended testes. By releasing the spermatic vessels and vas deferens, the testicle is fixed by tension-free drainage and the sphincter is ligated at a high level.^7^

Available surgical methods can be divided into OO and LO. Although OO is known for its effectiveness, this technique can result in a longer recovery time and the surgical incision is long, which may leave visible scarring.^8^ LO technology is a more recent technique which is minimally invasive, has fast postoperative recovery, and reduces scarring. However, LO requires greater technical experience as the abdominal wall must be punctured, which is a relatively difficult, but highly, procedure.^9^

At present, it is still controversial whether LO or OO is the first choice for the treatment of palpable undescended testes. This study will compare the clinical outcomes of the two surgical methods to provide more evidence for clinical practices.

## METHODS

The records of 76 children with palpable undescended testes treated in Zaozhuang Municipal Hospital from June 2019 to January 2021 were selected in this observational retrospective study. Pre- and post-operative ultrasound examinations were performed by pediatric surgeons. There were 62 unilateral and 14 bilateral cases. The patients ranged in age from two to eight years old, with an average of 4.5±1.4yrs. The duration of palpable undescended testis was 1-7 years, with an average of 3.4 ± 1.3yrs. Patients who received OO were set as Open-group (n=33), while patients received LO were set as Laparoscopic-group (n=43). Patients required staged operations in the Open-group and the Laparoscopic-group were 4 and 2, respectively. There was no significant difference between the two groups in patient characteristics (p>0.05, Table-I).

**Table-I:** Patients characteristics [n (%),*χ̅*±*S*].

Group	Number of cases (n)	Age (years)	Duration of palpable undescended testis (years)	Disease location (n)	

				Unilateral	Bilateral
Open-group	33	4.54±1.42	3.57±1.30	28	5
Laparoscopic-group	43	4.39±1.36	3.35±1.31	34	9
*t*/*χ^2^*	-	0.467	0.753	0.415	
p-Value	-	0.642	0.454	0.519	

### Inclusion Criteria:

- Palpable undescended testes confirmed by physical examination and B-ultrasonic examination by more than two pediatric surgeons,
- No possibility of spontaneous decline of undescended testes,
- No history of inguinal surgery,
- Complete medical records.

### Exclusion criteria:

- Testicular atrophy and absence,
- Umbilical infection,
- Other serious basic diseases and organ dysfunction,
- Other urinary system diseases.

### Ethical Approval:

This study was approved by the medical ethics committee of our hospital (No.: ZZMH10; Date: 2022-07-21).

### Open orchiopexy:

An incision, 2-3cm in length, was made at the transverse stria of the groin on the affected side. The anterior wall of the inguinal canal was exposed and opened to determine the location of the undescended testes. The vas deferens and spermatic cord vessels were then exposed, and high ligation of the hernia sac was performed. The vas deferens and spermatic cord were loosened, and the testicular lead was cut off. A tunnel from the incision to the scrotum was created, the skin at the bottom of the scrotum was cut off. The intima of the subcutaneous tissue was properly expanded, and the scrotal incision was held open with vascular forceps to allow the testis to be moved into the bottom of the scrotum along the expansion tunnel. The scrotum was fixed outside the sarcolemma, the incision was treated and the operation was completed.

### Laparoscopic orchiopexy:

The patient was supine, with their feet raised higher than their head. A 5mm trocar and CO2 pneumoperitoneum were placed in the umbilical incision to create the endoscopic monitoring viewport. A 5mm or 3mm trocar was placed on both sides of the umbilical incision, along with electrocoagulation hooks, separating forceps, and scissors. The lateral peritoneum was opened to fully free the spermatic cord blood vessels and vas deferens. The affected testes were drawn to the contralateral inner ring mouth without tension. If the testes were located in the inguinal canal, the peritoneum of the inner ring mouth was cut, and the spermatic cord blood vessels and vas deferens were loosened.

An incision of 1.0cm was made at the bottom of the scrotum, the subcutaneous space was passively separated, through the scrotum incision from the inner ring mouth of the peritoneum. The tunnel was expanded, and the testicle was moved along the tunnel without torsion into the scrotal sarcolemma space, sutured and fixed. The incision was treated, and the operation was completed. The follow-up included 76 children for 18 months post-operation. All children were followed up. Operation time, intraoperative bleeding, first ambulation time, hospitalization time, early and long-term complications were recorded for both groups.

Early complications included incision infection and scrotal hematoma, and long-term complications included incision hernia, testicular retraction and testicular atrophy. Testicular atrophy refers to loss of volume ≥ 50% after orchiopexy.^10^ Testicular growth rate and testicular volume at 18 months post-operation were also recorded. The criteria for testicular growth were: the testicles were located at the bottom of the scrotum with good elasticity, ultrasound examination found normal blood supply,^11^ testicular volume = testicular thickness × width × length.^12^

### Statistical analysis:

Referring to previous studies, we assumed that the postoperative atrophy rates would be around 0.6%-16.4% and 10.8%-28.3% in the Laparoscopic-group and the Open-group, respectively. The level of significance was at a two-sided a level of 0.05 with a power of 80% and potentially 10% incomplete data. At least 57 patients would be needed for the study.^13^-^15^ The statistical analysis software SPSS 22.0 was used for data analysis, with [n (%)] represents the counting data, the χ2 inspection was used for comparison between groups, with (*χ̅*±*S*) referring to measurement data. The two independent sample t-test was used for comparison between groups, and *p*<0.05 was considered statistically significant.

## RESULTS

Operation time, intraoperative bleeding, first time out of bed activity time and hospital stay in the Laparoscopic-group were less than those in the Open-group (*p*<0.05, Table-II). Regarding surgical complications, one patient in the Laparoscopic-group developed a scrotal hematoma, and the short-term complication rate was 2.27%. In the Open-group, three patients developed an incision infection, two patients developed scrotal hematoma, and the short-term complication rate was 15.15%. Regarding long-term complications, one patient in the Laparoscopic-group developed testicular retraction, one patient developed testicular atrophy, and the long-term complication rate was 4.65%.

**Table-II:** Surgery-related indicators (*χ̅*±*S*).

Group (n)	Operation time (minute)	Intraoperative blood loss (ml)	First time out of bed (day)	Hospital stays (day)
Open-group (n=33)	63.30±11.53	17.00±3.97	5.18±1.49	9.96±2.46
Laparoscopic-group (n=43)	52.11±8.31	9.09±2.22	3.72±1.33	6.95±1.69
t	4.914	10.258	4.501	6.020
p-Value	<0.001	<0.001	<0.001	<0.001

In the Open-group, one patient developed testicular retraction, and the long-term complication rate was 3.03%. Overall, the short-term complication rate in the Laparoscopic-group was lower than in the Open-group (*p*<0.05), with no significant difference in long-term complications between groups (*p*>0.05) Table-III. Following 18 months post-operation there was good testicular growth in the Laparoscopic-group, 97.67% (42/43), which was not significantly different from 96.97% (32/33) in the Open-group (χ2=0.036, *p*=0.849). The testicular volume of the Laparoscopic-group was 0.59 ± 0.14ml, which was not significantly different from the Open-group (t =0.335, *p* =0.739).

**Table-III:** Incidence of surgical complications [n (%)].

Group	Number of cases (n)	Short-term complications	Long-term complications
Open-group	33	5 (15.15)	2 (4.65)
Laparoscopic-group	43	1 (2.27)	1 (3.03)
χ^2^	-	4.224	0.687
p-Value	-	0.040	0.407

## DISCUSSION

The results presented here show that LO was superior in operation time, intraoperative bleeding, first out of bed activity time and hospitalization time when compared to OO in the treatment of palpable undescended testes. There was no difference in the incidence of complications, growth rate and testicular volume between the two surgical methods 18 months post-operation. Similarly, work by Feng X et al^16^, suggests that LO is less traumatic than OO in treating undescended testes. A retrospective study of 291 children^17^ compared LO with traditional inguinal incision orchiopexy (TIO) and found that LO had a significantly shorter mean operation time and postoperative normal activity time and a higher success rate compared with TIO. We believe this is because laparoscopy can magnify the surgical field of view by 2-3 times and can realize close tracking of the surgical field of view, making it easier to perform operations, which in turn can improve operational efficiency, shorten operation time, avoid unnecessary injuries, and reduce postoperative complications.

A meta-analysis comparing LO and OO resulted in no significant differences in the success rate and long-term complications, but LO was safer and more effective.^18^ Although the testicular retraction rate is low post-operation, it is easy to damage the spermatic cord vessels when performing inguinal surgery, hernia sac dissection or other similar surgeries. These operations can be long in duration, with larger intraoperative bleeding which could increase postoperative recovery time.^19^

LO, as a minimally invasive surgery, has been the gold standard for the treatment of nonpalpable undescended testes. This allows the operation to be performed under the direct vision of the laparoscope. Additionally, a trocar puncture is used to create a tunnel to guide the testes to bottom of the scrotum. The operation time is short with less intraoperative bleeding, which can further reduce any surgical trauma and accelerate the postoperative recovery process of patients.^20^

OO requires a larger incision, dissection of the inguinal canal and results in a longer operation time, increasing the risk of infection and scrotal hematoma.^8^ In this study, the short-term complication rate following LO was lower than OO (*p*<0.05), with no differences in long-term complications (*p*>0.05). Further, 18 months post-operation showed no difference in testicular growth and testicular volume between groups (*p*>0.05).

Kojima T et al^21^ examined the efficacy of LO and OO in the treatment of undescended testes. Their conclusions confirm the results presented here, in that LO and OO both result in successful testicular growth, however LO can further reduce the surgical trauma and short-term surgical complications. Interestingly, this is evidence which shows no difference in the incidence of postoperative complications between LO and OO in the treatment of undescended testes (*p*>0.05), although these results may be related to the small sample size selected in this study, and may not be representative of all children with undescended testes.^22^

In clinical practice, undescended testes can be divided into three types: abdominal, inguinal and absent ones.^23^ Typically, the first choice for intraperitoneal treatment is LO, while there is some debate surrounding whether LO should be used to treat inguinal and absent ones. Some surgeons believe that OO has a more significant therapeutic effect when used with these two types of undescended testes, although LO has been recommended by some.^24^

In the treatment of palpable undescended testes, both LO and OO have their own advantages. However, although there were no significant differences in long-term complication rate and postoperative testicular growth, LO may be preferred because of its shorter operation time, less intraoperative bleeding, and faster postoperative recovery. Therefore, in clinical practice, the operation method can be reasonably selected according to the specific diagnosis and medical circumstances of the pediatric patient.

### Limitations:

In this study, 76 children were included who were followed up for 18 months. This is a relatively small sample size, with a limited follow up duration, and the results do not fully represent all children with undescended testes may affect the objectivity and comprehensiveness of the conclusions. Future studies involving more large-scale multicenter locations, with greater sample sizes needed, with a longer follow up, to enhance the evaluation of the clinical effects of LO and OO are in the treatment of undescended testes.

## CONCLUSION

In the treatment of palpable undescended testes, we found no significant differences in the long-term complication rate and postoperative testicular growth and testicular volume of patients treated with LO and OO. Shorter operation time, less intraoperative bleeding and rapid recovery time however have been noted with LO.

### Authors’ contributions:

**ZC** conceived and designed the study.

**YC and YH** collected the data and performed the analysis.

***ZC*** was involved in the writing of the manuscript and is responsible for the integrity of the study.

All authors have read and approved the final manuscript.
